# Efficacy of Pulsed 405-nm Light-Emitting Diodes for Antimicrobial Photodynamic Inactivation: Effects of Intensity, Frequency, and Duty Cycle

**DOI:** 10.1089/pho.2016.4179

**Published:** 2017-03-01

**Authors:** Jonathan B. Gillespie, Michelle Maclean, Martin J. Given, Mark P. Wilson, Martin D. Judd, Igor V. Timoshkin, Scott J. MacGregor

**Affiliations:** ^1^ Department of Electronic & Electrical Engineering, The Robertson Trust Laboratory for Electronic Sterilisation Technologies, University of Strathclyde, Glasgow, Scotland, United Kingdom.; ^2^ Department of Biomedical Engineering, The Robertson Trust Laboratory for Electronic Sterilisation Technologies, University of Strathclyde, Glasgow, Scotland, United Kingdom.; ^3^ Department of Electronic & Electrical Engineering, High Voltage Technologies, University of Strathclyde, Glasgow, Scotland, United Kingdom.

**Keywords:** antimicrobial, 405 nm light, light-emitting diodes, pulsed light, *Staphylococcus*

## Abstract

***Objective:*** This study investigates possible advantages in pulsed over continuous 405-nm light-emitting diode (LED) light for bacterial inactivation and energy efficiency. ***Background:*** Alternative nonantibiotic methods of disinfection and infection control have become of significant interest. Recent studies have demonstrated the application of systems using 405-nm LEDs for continuous disinfection of the clinical environment, and also for potential treatment of contaminated wounds. ***Methods:*** Liquid suspensions of 10^3^ colony-forming units/mL populations of *Staphylococcus aureus* were subject to pulsed 405-nm light of different frequencies, duty cycles, and intensities and for different lengths of time. ***Results:*** Pulsed exposures with the same average irradiance of 16 mW/cm^2^ and varying duty cycle (25%, 50%, 75%) showed very similar performance compared with continuous exposures, with 95–98% reduction of *S. aureus* achieved for all duty cycles. The pulsing frequency was varied in intervals from 100 Hz to 10 kHz and appeared to have little effect on antimicrobial efficacy. However, when comparing pulsed with continuous exposure, an improvement in inactivation per unit optical energy was achieved, with results showing an increase of approximately 83% in optical efficiency. ***Conclusions:*** These results suggest that under pulsed conditions, a lower energy consumption and lower perceived brightness could be achieved, thus potentially providing improved operating conditions for medical/infection control applications without compromising antimicrobial efficacy.

## Introduction

The antimicrobial effects of violet–blue light have been reported using wavelengths in the region of 400–420 nm, with peak inactivation demonstrated at 405 nm.^1^ Absorption of this light by endogenous porphyrins results in the production of reactive oxygen species, including H_2_O_2_ and singlet oxygen, leading to oxidative damage and cell death.^2–5^ Compared with ultraviolet (UV) radiation, 405-nm light offers reduced germicidal efficiency; however, it can be used at levels that are lethal to microorganisms,^1,6–11^ without affecting exposed mammalian cells.^12–14^

Recent studies have reported the use of 405-nm light for continuous decontamination of hospital environments,^15,16^ and its potential use for a range of medical and infection control applications has been demonstrated, including wound decontamination (with no adverse effect on wound healing)^17^ and sterilization of tissue matrices such as collagen.^18^

As the germicidal efficacy of 405-nm light is significantly lower than that of UV light, it is important to investigate methods by which both the germicidal and operational efficacy of 405-nm light could be improved. Pulsed operation of UV light-emitting diodes (LEDs) has reportedly resulted in improved antimicrobial efficacy^19,20^; therefore, it is of interest to investigate whether the same principle can be applied to 405-nm light.

The application of 405-nm light for continuous environmental decontamination^15,16^ requires lighting levels to be comfortable for room occupants. Therefore, any improvements in either electrical or antimicrobial efficiency could allow for the use of a lower radiant light intensity, or apparent photometric light intensity, and thus a more esthetically acceptable light output.

The present study compares the antimicrobial and operational efficacy of pulsed and continuously operated 405-nm LEDs using the hospital pathogen, *Staphylococcus aureus*. The effect of varying the peak intensity and dose of 405-nm LED light was investigated, in addition to varying the duty cycle (25%, 50%, 75%) and frequency (100 Hz–10 kHz), to determine whether any pulsed regime could deliver improved antimicrobial efficacy or electrical/optical efficiency when compared with existing continuous regimes.

## Methods

### Bacterial preparation

*S. aureus* NCTC 4135 (National Collection of Type Cultures, Colindale, United Kingdom) was cultured for 18 h in 100 mL nutrient broth (Oxoid Ltd., United Kingdom) under rotary conditions (37°C, 120 rpm). The culture was centrifuged at 3939 *g* for 10 min at 20°C, resuspended in 100 mL phosphate-buffered saline, and serially diluted to 10^3^ colony-forming units per milliliter (CFU/mL).

### Electronic circuits

**FIG. 1.**
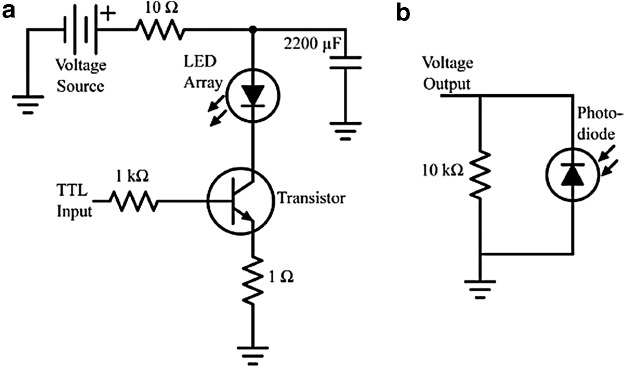
The electronic circuits used for pulsing and light measurement. **(a)** Circuit used to provide the input power, pulsed or continuous, to the LED array for bacterial exposure. **(b)** Circuit used to measure the voltage output from the photodiode to monitor the optical output of the LED array. LED, light-emitting diode.

The pulsing circuit used is shown in Fig. 1a.

The frequency and duty cycle of the optical pulses were set by a 5 V TTL signal from a pulse generator. The duty cycle is the percentage of a period of a waveform during which the signal is high or, in this case, the LED array is on: given by Equation [1], with pulse width, *τ*, and pulse period, *T*.

\begin{align*}
Duty \ Cycle = \frac { \tau }  { T } \times 100 \% 
\quad\quad\quad\quad [1]
\end{align*}

The circuit was used to operate the LED array either continuously (100% duty cycle) or pulsed (25%, 50%, 75% duty cycles), depending upon the TTL input. The LEDs were powered using a voltage in the range of 30–40 V.

The optical output was captured by a photodiode (BPW34 B, OSRAM) used in conjunction with a resistor (Fig. 1b). This setup provided an arbitrary reading of the optical output in millivolts to ensure the optical output remained constant. Absolute intensity was recorded using a radiant power meter (Model-70260; Oriel Instruments) and photodiode detector (Model-1Z02413; Ophir), calibrated at 405 nm, and a spectrometer (Ocean Optics HR4000). Voltage and current waveforms were viewed using a Tektronix TDS 2024 digital storage oscilloscope.

### Four hundred five-nanometer light source arrangement

**FIG. 2.**
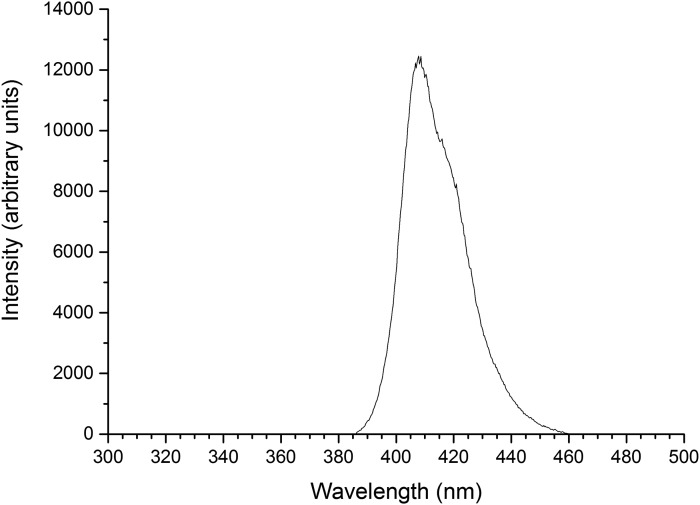
Spectral output of the 405-nm LED array used for bacterial exposure.

The light source used was the 405-nm ENFIS Innovate UNO 24 (PhotonStar Technology Ltd., United Kingdom): an array of 24 × 405-nm LEDs, with a typical central wavelength of 405- and 16-nm FWHM (Fig. 2).

**FIG. 3.**
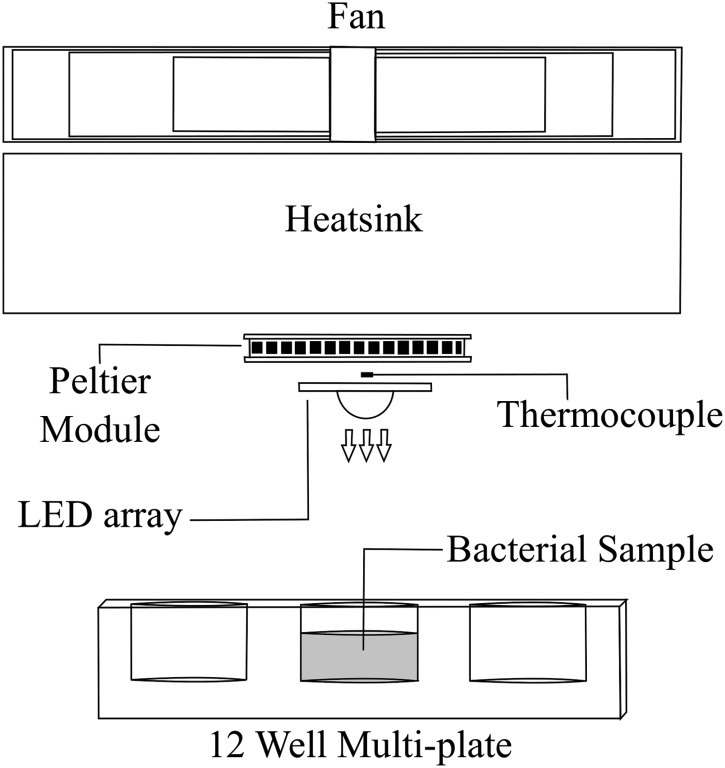
Cross-sectional view of the experimental setup showing the LED array system and the layout of the thermal management components used for the exposure of bacterial samples.

A thermal adhesive was used to uniformly interface the heat sink, Peltier module, LED array, and thermocouple (Fig. 3). Four mL 10^3^ CFU/mL suspension was positioned 10 cm below the light source and exposed to increasing durations of light. Samples were taken at regular intervals and surviving populations enumerated. Sample temperature was also recorded. This setup was the same for each experiment, with the only variation being the light treatment regimes and sampling times (detailed in Sections 2.4–2.6). For all experiments, current and voltage were monitored continuously; the bulk temperature of the LED array was measured and controlled throughout; and the photodiode was employed to monitor the light level.

### Investigating the effects of duty cycle and peak intensity variation

The peak intensity and duty cycle were varied to determine their effect on inactivation. The time, temperature, frequency, and overall dose of each exposure were kept constant at 120 min, 24°C, 1 kHz, and 115 J/cm^2^, respectively. During continuous exposure, the irradiance was kept constant at 16 mW/cm^2^. For pulsed exposure, the peak irradiance was varied to maintain a constant overall dose, with the peak irradiance for each experiment being 64, 32, 21, and 16 mW/cm^2^ for the duty cycles 25%, 50%, 75%, and 100% (continuous), respectively.

### Investigating the effects of frequency variation

Bacteria were exposed to pulsed light for 60 min at a 50% duty cycle, with a constant intensity, dose, and bulk LED temperature of 24 mW/cm^2^, 86 J/cm^2^, and 24°C, respectively. The pulse frequency was varied across five frequencies: 100 Hz, 500 Hz, 1 kHz, 5 kHz, and 10 kHz.

### Investigating pulsed-delivered dose and resulting bacterial inactivation

A set of experiments was undertaken to investigate whether the bacterial inactivation achieved by pulsed light was proportional to the dose delivered.

Exposures were 40 min long with a peak intensity of 29 mW/cm^2^ and a frequency of 1 kHz, but each with a different dose, which varied directly with the duty cycle. The doses were 17.4, 34.8, 52.2, and 69.6 J/cm^2^ for the 25%, 50%, 75%, and 100% duty cycles, respectively.

Optical efficiency (bacterial inactivation in CFU/mL achieved per unit of optical energy applied per cm^2^) was used to measure performance. The overall power consumption was measured using a VIVID power meter (model: GT-PM-04), and the overall energy consumption was calculated.

### Assessment of bacterial inactivation

Bacterial samples (100–500 μL) were taken at the start, during, and end of the treatment periods. Control samples, exposed to normal laboratory lighting, were used in all experiments. Samples were spread-plated onto nutrient agar (Oxoid Ltd), incubated at 37°C for 24 h, and surviving populations enumerated.

The experimental data represent averages of a minimum of triplicate independent experimental results, measured in duplicate (*n* ≥ 6), with standard deviations. Data sets were analyzed using one-way analysis of variance and Tukey's tests with OriginPro 9.0.0 statistical software, with significant differences accepted at *p* ≤ 0.05.

## Results

### Investigating the effects of duty cycle and peak intensity variation

**FIG. 4.**
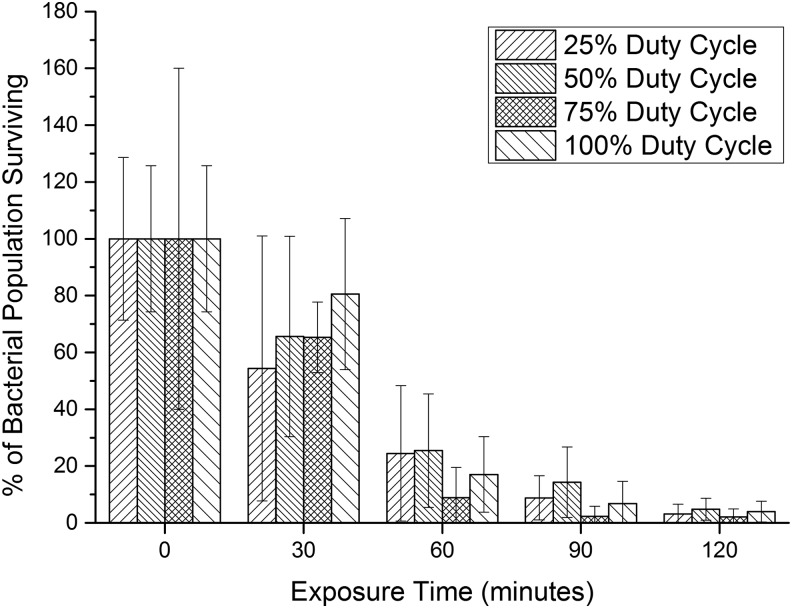
Comparison of the inactivation effects of pulsed 405-nm LED light exposures with varying duty cycles (25%/50%/75% duty cycles) and continuous LED light exposures (100% duty cycle) on bacterial suspensions of *Staphylococcus aureus*. Irradiance was held constant at 16 mW/cm^2^. Each bar is a mean value (*n* ≥ 6) ± SD, all given in percentage in relation to the control. SD, standard deviation.

Figure 4 shows how the method of delivery of a constant dose of 405-nm light affects the bacterial inactivation achieved.

Dose and exposure time were constant in each experiment, and peak intensity and duty cycle varied. For 120-min exposures, a relatively uniform reduction of ∼96% (2-log_10_ CFU/mL) in the bacterial population was achieved for all duty cycles. Although fluctuations were observed, no significant difference (*p* ≥ 0.05) was found between any of the surviving bacterial populations at any specific time point. Control samples showed no significant change over the course of the experiments (*p* ≥ 0.05). These results suggest that with the same dose applied over the same exposure time (while varying duty cycle and peak intensity), the antimicrobial efficacy is not affected, either beneficially or adversely, when the light is applied in a pulsed rather than continuous regime.

Similar studies using pulsed UV light have found differing results, and although not directly comparable with the present study due to the differing wavelengths and inactivation mechanisms involved, it is interesting to discuss the results with respect to the potential benefits of pulsed light regimes. In a study by Wengraitis et al.,^20^ *Escherichia coli* biofilms exposed using UV-C LEDs showed a change in sensitivity with duty cycle. Results showed a fourfold increase in sensitivity as the duty cycle dropped from 90% to 10%, suggesting a strong correlation between duty cycle and bacterial inactivation.^20^

Increased inactivation of *E. coli* biofilms with pulsed application of UV-A LEDs was shown by Li et al.,^19^ with a reduction of 98.7% using a 75% duty cycle, compared with ∼87% by continuous exposure. While it appears that the doses delivered in each regime may not have been constant, the results do indicate that duty cycle may have an effect on inactivation.

In these UV studies,^19,20^ the exposure time, rather than the peak intensity, was altered to keep the dose constant. The longer exposure times could possibly have resulted in increased inactivation through the action of radicals generated in the bacterial suspensions. All exposures in the present study were run for the same time in an effort to avoid this effect.

### Investigation into the effects of frequency variation

**FIG. 5.**
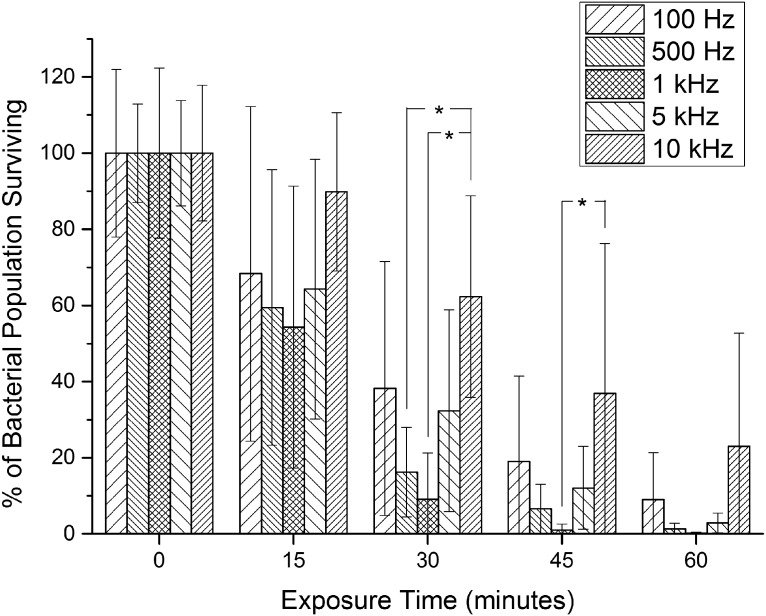
A comparison of the inactivation effects of 405-nm LED light exposure with varying frequencies (100 Hz–10 kHz) on bacterial suspensions of *S. aureus*. Each bar is a mean value (*n* ≥ 6) ± SD, all given in percentage in relation to the control. *Significant difference (*p* < 0.05) between the two points indicated by either side of the asterisk bracket.

Bacterial inactivation kinetics achieved using five different frequencies, ranging from 100 Hz to 10 kHz, are shown in Fig. 5.

The duty cycle was fixed at 50%, and the average irradiance for each of the exposures was 24 mW/cm^2^. There was divergence between the kinetics at 10 kHz and those at lower frequencies. The results show successful inactivation across the range of frequencies, varying from ∼77% up to 99.8% reductions after 60-min exposures. The kinetics at 10 kHz demonstrated a statistically slower rate after 30 and 45 min; however, there was no significant difference (*p* ≥ 0.05) between the populations after 60 min.

For all tested frequencies, there appeared to be no difference in the total inactivation achieved. The Stark–Einstein Law states that light absorbed need not necessarily result in a photochemical reaction, but if it does, only one photon is required for each molecule effected.^21^ This suggests that each porphyrin molecule only needs to absorb one photon of 405-nm light. If more than one porphyrin molecule is present inside the cells, it could be that a certain number of these porphyrins must absorb photons to cause complete inactivation of the cell. Hence, with a short pulse of photons, it may be the case that not enough porphyrins are being excited, which could result in slower cell death or a reduced rate of inactivation.

Li et al.^19^ carried out similar experiments on *Candida albicans* and *E. coli* biofilms using pulsed UV-A LEDs. From a frequency range of 0.1 Hz–1 kHz, 100 Hz was found to be significantly (*p* < 0.01) more effective for microbial inactivation.

A study using UV-C LEDs^20^ compared different duty cycles and frequencies, but the exposure time rather than the peak intensity of the light was varied to keep the dose constant. Varying frequencies in the range 0.5–100 Hz were found to have an effect on inactivation, but there was no clear trend showing whether higher or lower frequencies were more or less effective for the various regimes.

Wengraitis et al.^20^ used frequencies from 0.1 to 100 Hz and Li et al.^19^ used 0.1–1000 Hz, with varying degrees of resolution. The frequencies in the present study were higher, spanning from 100 Hz to 10 kHz, with this range chosen bearing in mind possible practical antimicrobial applications. One of the main benefits of the use of 405-nm light for disinfection is that it can be used safely in the presence of humans and thus it would be desirable to design a system with comfort in mind, that is, to utilize a higher-frequency pulsing regime that appeared continuous to the human eye.

### Investigating the relationship between pulsed-delivered dose and bacterial inactivation

Referring to Table 1, 25%, 50%, and 75% duty cycles appear to show an increase in average optical efficiency when compared with continuous irradiation, with the 25% duty cycle showing >80% increase in optical efficiency.

**Table 1.** The Experimental Results Showing the Relationship of a Pulsed Dose of 405-nm Radiation and the Bacterial Inactivation Achieved

*Duty cycle (%)*	*Unexposed sample (CFU/mL)*	*Exposed sample (CFU/mL)*	*Percentage reduction (%) ± std deviation (%)*	*Dose (J/cm*^2^*)*	*Average optical efficiency*^a^*[(CFU/mL)/(J/cm*^2^*)] ± std deviation*	*Energy consumption (kJ)*
25	2272	1161	48.9 ± 22.6	17.4	55.7 ± 15.8	87.9
50	2246	655	70.8 ± 27.3	34.8	41.9 ± 27.6	112.8
75	2172	273^*^	87.4 ± 35.7	52.2	36.1 ± 12.7	132.8
100	2167	24^*^	98.9 ± 39.2	69.6	30.4 ± 1.5	174.0

In addition, the power consumption is measured, allowing for a comparison of optical efficiency and electrical energy consumption.

^a^ Bacterial inactivation per unit of optical energy per cm^2^ applied.

^*^ Bacterial populations that were significantly different from the population at 25% duty cycle (*p* < 0.05).

CFU, colony-forming units.

These results indicate that inactivation is not entirely dose dependent. Statistical analysis showed only two significant differences in the exposed populations: the final populations exposed to the 25% duty cycle were different from those of the 75% duty cycle (*p* = 0.028); and the final populations exposed to the 25% duty cycle were different from those of the continuous exposure (*p* = 0.004). Hence, the inactivation achieved through continuous exposure is not significantly different from that achieved through 50% or 75% duty cycle exposure, meaning that the 50% and 75% duty cycles result in the same level of inactivation for 50% and 75% of the dose, respectively.

With this in mind, and considering the energy consumption, the 50% duty cycle used ∼60 kJ less energy than the continuous exposure, but statistically achieved the same degree of inactivation; likewise, the 75% duty cycle used ∼41 kJ less energy than the continuous regime for the same level of inactivation. At 25% duty cycle, there was a significant difference between the bacterial reductions compared with the continuous regime, but the energy consumption is almost 50% less.

Differing from the results in Table 1, initial experiments showed no improvement in antimicrobial efficacy. These results may have been masked in initial experiments by the simultaneous change in peak intensity and duty cycle to keep the overall dose constant. The results of the dose-dependency experiments agree with those of Wengraitis et al.,^20^ who also found an increase in both energy efficiency and sensitivity (equivalent of optical efficiency) with low duty cycles of 10% and 25%, although with UV-C LEDs. Li et al.^19^ found that pulsed exposure using UV-A LEDs resulted in more effective inactivation. Although the results agree, exposure times were changed in both UV studies, prohibiting direct comparison with the present study.

Considering the increased optical efficiency observed, it may be that there is a minimum time required for complete inactivation of a bacterial cell to occur, regardless of light intensity. Assuming that there are multiple porphyrins capable of photon absorption per cell, there could be a point at which the absorption of more photons would have little effect on the inactivation mechanism already in progress. Pulsing light with on/off periods of exposure could therefore be more efficient, with optimal efficiency being achieved if the pulse width could be matched with the inactivation time constant. This would possibly allow for photons to reach other cells that were perhaps shielded previously.

This idea also contributes to the thought that dose might not be the only factor in inactivation efficacy and could explain why lower duty cycles show more efficient inactivation in terms of optical efficiency and energy consumption. The off period may give time for absorption of light and generation of radicals to occur and cause damage to the cells, with less photons being absorbed unnecessarily.

In physical terms, higher intensity means higher photon flux, meaning more photons per second reaching the sample area.^22^ The energy of the individual photons remains unaffected as given by Planck's relation.^21^ It seems intuitive that there will come a point at which the bacterial sample is saturated and cannot absorb any additional photons, thus the cumulative energy effect is lost. This would suggest that although there may be a range of intensities at which inactivation will be relatively dose dependent, the relationship between intensity, dose, and inactivation will likely be nonlinear.

## Conclusions

This study has indicated that pulsing of 405-nm LED light, while maintaining the same dose over the same period of time, or varying the frequency, has similar performance when compared with the continuous exposure in terms of antimicrobial efficacy.

Although the antimicrobial efficacy is unchanged, pulsed 405-nm LED light appears to be more optically efficient. The maximum average optical efficiency increased by ∼80% with a 25% duty cycle compared with continuous exposure. The energy consumption showed a downward trend as the duty cycle decreased, but for the 50% and 75% duty cycles, the same level of bacterial inactivation was achieved as for the continuous exposure, with lower energy consumption.

These results align well with the application of 405-nm light for practical infection control treatments. With particular reference to its use for continuous environmental decontamination, if running an LED-based lighting system at a 50% duty cycle, the same amount of microbial inactivation could be achieved with ∼35% less electrical energy consumed, and the light would appear less bright than if it were operated continuously. This would help improve the esthetics and acceptability of antimicrobial room lighting systems, with enhanced 405-nm light output giving lower perceived brightness and the added benefit of lower electrical energy consumption, which could lead to substantial savings given that the application requires the lights to be operated for extended periods.
